# Supplementary material for: Generation and Characterization of Stable Small Colony Variants of USA300 Staphylococcus aureus in RAW 264.7 Murine Macrophages
Source: Antibiotics (Basel). 2024 Mar 16;13(3):264. doi: 10.3390/antibiotics13030264 (PMC10967292; doi:10.3390/antibiotics13030264)
Supplement: Supplementary file 1 [file antibiotics-13-00264-s001.zip › antibiotics-2869538-supplementary.pdf]

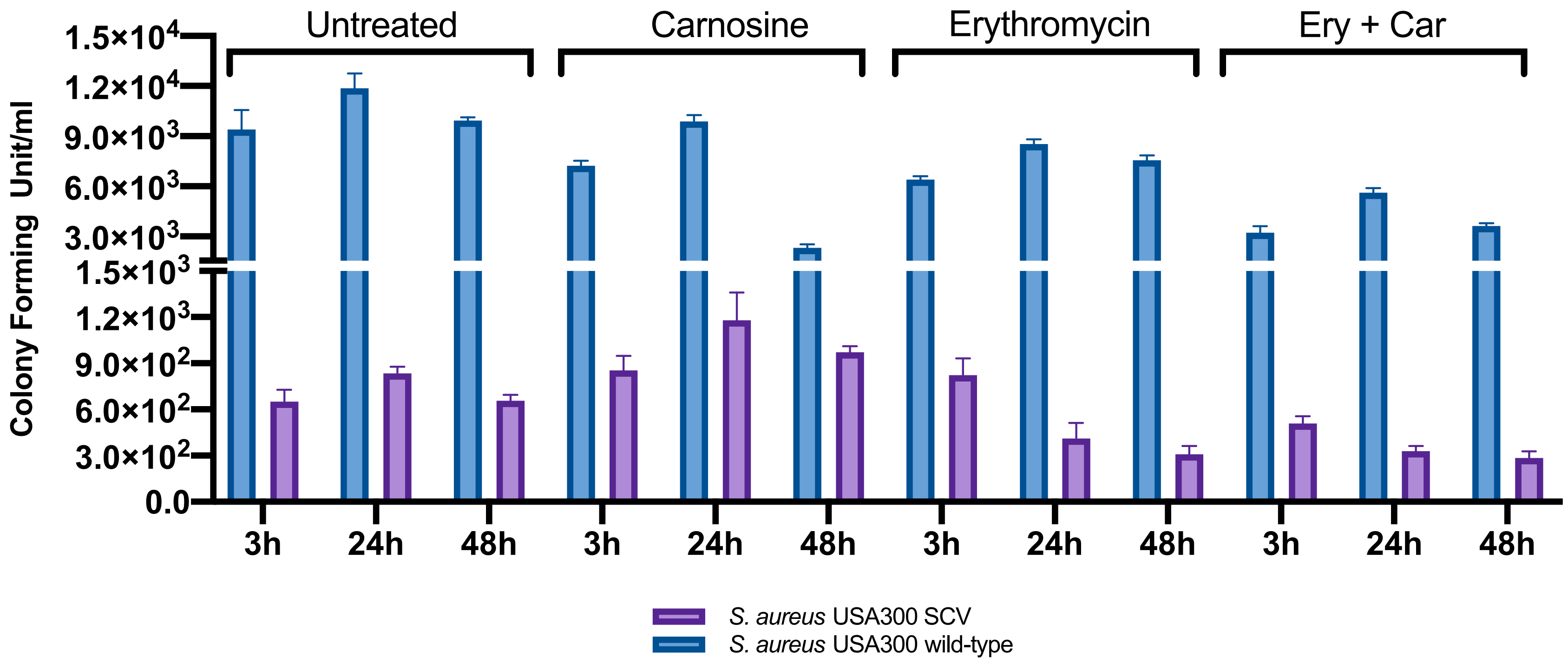

Figure S1: Colony count (CFU/ml) from all experimental conditions at 3, 24 and 48h p.i.

Table S1: Aminoglycoside MIC values of S. aureus ATCC BAA-1556_Catania and its derivative SCVs

	USA 300	
	WT	SCV
AMIKACIN	4 mg/L	96 mg/L
GENTAMYCIN	0.5 mg/L	4 mg/L
TOBRAMYCIN	0.5 mg/L	6 mg/L
KANAMYCIN	1.5 mg/L	16 mg/L

Table S2. Resistance and virulence profiles of ATCC BAA-1556 *S. aureus* WT and its derivative stable SCV.

RESISTANCE AND VIRULENCE PROFILES	
Resistance genes	*mecA, gene encodes methicillin resistance in staphylococci; fosB, gene that leads to the resistance of fosfomycin; mepA, gene encodes MepA efflux pumps; arlS, protein histidine kinase for ArlR; mepR, gene for upstream repressor of MepA; tetK, gene encodes a tetracycline efflux pump; ermC, ribosomal RNA methyltransferase; lmrS, secondary active transporters; arlR, response regulator for norA; norA, multidrug efflux pump; tet38, tetracycline efflux pump; mepA, gene for efflux pump protein; mgrA, regulator for norA, norB, and tet38.Virulence genes: hla, alpha-hemolysin; hlgA beta-hemolysin; hld, delta-lysin; gamma-hemolysin A; hlgB, gamma-hemolysin B; hlgC; gamma-hemolysin C; aur, aureolysin; ebp, elastin-binding protein; IcaR-IcaA-IcaD-IcaB-IcaC-IsdB-IsdA-IsdC-IsdD, iron metabolism and acquisition; isdE-isdF, heme metabolism srtB, isdG, lip, geh, map, lukS/F PV, Panton Valentine leukocidin encoding factors; scn, staphylococcal complememnt inhibitor; sdrD, serine-aspartate repeat-containing protein D; sdrE, serine-aspartate repeat-containing protein E; sspB, staphylococcal serin-protease B; sspC, staphylococcal serin-protease C; sak, staphylokinase; esxA, staphylococcal protein secretion system A; esxB, staphylococcal protein secretion system B; esaA, staphylococcal secretion system component A; essA, staphylococcal secretion machinary protein A; essB, staphylococcal secretion machinary protein B; essC, staphylococcal secretion machinary protein C; vWbp, von Willebrand factor-binding protein.*
Virulence genes	*hla, alpha-hemolysin; hlgA, beta-hemolysin; hld, delta-lysin; gamma-hemolysin A; hlgB, gamma-hemolysin B; hlgC, gamma-hemolysin C; aur, aureolysin; ebp, elastin-binding protein; IcaR-IcaA-IcaD-IcaB-IcaC-IsdB-IsdA-IsdC-IsdD, iron metabolism and acquisition; isdE-isdF, heme metabolism srtB, isdG, lip, geh, map, lukS/F PV, Panton Valentine leukocidin encoding factors; scn, staphylococcal complememnt inhibitor; sdrD, serine-aspartate repeat-containing protein D; sdrE, serine-aspartate repeat-containing protein E; sspB, staphylococcal serin-protease B; sspC, staphylococcal serin-protease C; sak, staphylokinase; esxA, staphylococcal protein secretion.*

Table S3: statistical insights of gene expression levels detected through qRT-PCR

***S.aureus* USA300 Vs *S.aureus* SCV**		Unpaired t test P value	P value summary	Significantly different (P < 0.05)?	95% confidence interval	R squared (eta squared)
Regulator	*sarA*	0,5214	ns	No	-1,058 to 1,520	0,229
	sigB	0,7005	ns	No	-0,4126 to 0,5075	0,08971
	agrA	0,1483	ns	No	-2,962 to 9,757	0,7255
Virulence	*psmA*	0,844	ns	No	-1,746 to 1,574	0,02434
	hla	0,0878	ns	No	-0,9560 to 6,173	0,8321
	hld	0,097	ns	No	-1,344 to 7,353	0,8155
Surface protein	*sdrE*	0,0459	*	Yes	0,07210 to 3,155	0,9103
Metabolic Pathways (TCA - Glycolysis)	*pdhA*	0,8241	ns	No	-1,769 to 1,573	0,03093
	fumC	0,3329	ns	No	-0,9176 to 1,683	0,445
	uhpt	0,0209	*	Yes	0,7451 to 3,307	0,9586
